# Application of a Cloud Model-Set Pair Analysis in Efficacy Assessment for Diabetic Ulcers

**DOI:** 10.1155/2019/8450397

**Published:** 2019-06-18

**Authors:** Le Kuai, Jia-qi Xing, Jing-ting Zhang, Xun-zhe Xu, Min-feng Wu, Ke-qin Zhao, Bin Li, Fu-lun Li

**Affiliations:** ^1^Department of Dermatology of Yueyang Hospital, Shanghai University of TCM, Shanghai 200437, China; ^2^Institute of Dermatology, Shanghai Traditional Chinese Medicine Research Institute, Shanghai 201203, China; ^3^Acupuncture and Manipulation College, Shanghai University of Traditional Chinese Medicine, Shanghai 201203, China; ^4^Shanghai Chinese and Western Medicine Hospital, Shanghai University of TCM, Shanghai 200082, China; ^5^Zhuji Research Institute of Connection Mathematics, Zhuji 311811, China; ^6^Department of Dermatology, Shaanxi Traditional Chinese Medicine Hospital, Xi'an 710003, China

## Abstract

Because treatment of diabetic ulcers includes various uncertainties, efficacy assessments are needed and significant. In previous studies, set pair analysis (SPA) has been applied to the efficacy assessments of traditional Chinese medicine (TCM) that pick out uncertainties related to the development and prognosis of disease. Optimized clinical protocols of SPA improve clinical efficacy. In the article, cloud model (CM) is employed to improve SPA, and a novel efficacy assessment method for a treatment of diabetic ulcers is proposed based on the cloud model-set pair analysis (CM-SPA). It is recommended to replace connection degree (CD) with cloud connection degree (CCD) that the efficacy assessment results are shown as normal clouds. Then, three diabetic ulcers patients treated with TCM made importance assessment by both CM-SPA and AHP based SPA. The comparison of assessment results shows that the CM-SPA is efficacious for the efficacy assessment of a treatment for diabetic ulcers and the results will be more scientific and accurate via CM-SPA.

## 1. Introduction

The refractory diabetic ulcer has confused patients for several years, especially in some developing countries. Studies show that 85% of patients suffer amputation [1]. Diabetic wound repair process is an extremely complex mechanism of signaling network which involves multiple factors such as tissues, cells, molecules, and genes. To cure the disease, many treatments were found and it is necessary to filter the best of them. Based on clinical validation, traditional Chinese medicine (TCM) has proved to be safe and curative effect in relieving pains of diabetic ulcers in recent years; furthermore, basic experiments have also proved part of mechanism on TCM in the treatment of diabetic ulcers [2–4]. However, it is hard to evaluate the exact efficacy because there are many uncertainties while healing. Efficacy assessment is widely used in the clinical evaluation. SPA could analyze fuzzy and uncertain factors which are integrated with TCM syndrome differentiation. In prophase researches, SPA is a powerful tool which could be used in systematic efficacy assessment for diabetic ulcers and improve clinical efficacy [5, 6]. The core of SPA is to ensure the weight of each assessment unit which needs to be further improved. Index weight could be valued in several ways for different purposes. At present, there are many methods that can be used to evaluate the curative effect of diabetic ulcer, such as risk index method and analytic hierarchy process (AHP). Fuzzy methods with AHP can help index weights more solid. Index weights calculated using the above methods will greatly influence the SPA results to evaluate efficacy assessment. Index weight is a subjective conclusion with multidirectional deviation because it is a reflection confirmed via professional decision. For that reason another way is introduced to calculate the uncertainty coefficient to make several decisions unify called cloud model (CM). CM is composed of droplets, and droplets arrange in disorder. A droplet is a qualitative unit represented in numerical value. Single droplet is of little importance, but more cloud droplets can reflect overall characteristics of qualitative concept. CM not only reflects the uncertainty of concepts in natural language, but also reflects the relationship between randomness and fuzziness, which constitutes a qualitative and quantitative mapping. It has been widely applied in various fields. Accelerated life test data [7], stability evaluation of rock slopes [8], topology optimization [9], risk assessment [10], image segmentation [11], and other fuzzy data analysis also require CM. Moreover, CM is advanced in process ambiguous linguistic data [12, 13].

Integrate above means expert judgment firstly input as the linguistic data to establish CM to confirm more scientific and reasonable index weight. The study is going to prove that basing cloud model-set pair analysis (CM-SPA), which is a more valid novel efficacy assessment method. In contrast to past studies, the weight calculated by the advanced method denoted as the cloud descriptors instead of constant values. In the next step, cloud weight further computes cloud connectivity (CCD) instead of SPA connectivity (CD). Finally, efficacy assessment of three diabetic ulcers patients after TCM treatment, respectively, made by CM-SPA and AHP based SPA are showed.

## 2. Method

### 2.1. SPA

SPA is one of the contact mathematics methods proposed by Zhao [14], which can integrate the certainty and uncertainty of problems into unified conclusions. SPA establishes the relationship mathematical model of certainty and uncertainty, and the value of N, S, P, F meets the equation N=S+P+F. The connection of pair set H is described as CD; an arithmetic expression for calculating the connection degree is as follows:

(1) $$\begin{array}{c} \varphi \left(\;H\;\right)=\frac{S}{N}+\frac{F}{N}i+\frac{P}{N}j=a+bi+cj \end{array}$$

Equation (1) establishes the basic SPA model. If ‘*i*' and ‘*j*' are certainly valued, the CD could be calculated. Theoretically, we divide the discrepancy set into ‘m' sets and the pattern should be expanded to general types named* m*-element model. The calculation formula is as follows:

(2) $$\begin{array}{c} \varphi =a+\overset{m-2}{\underset{n=1}{\sum }}b_{n}i_{n}+cj \end{array}$$

In (2) ‘*b*_*n*_' denotes the discrepancy degree of ‘m' sets and ‘*i*_*n*_*'* denotes the discrepancy coefficient of each grade.

Assume index k is any of indices. For the CD of index k, value of coherence degree, distinction degree, and contrast degree is set as ‘1'or ‘0', depending on its assessment value vest in effective grade or not. Usually, the main emphasis of SPA is how to ensure the index weight. In the ordinary way, short-cut process of constant was used for the index weight. As a result, the total CD could be calculated by

(3) $$\begin{array}{c} \varphi =\overset{r}{\underset{k=1}{\sum }}\varphi_{k}\omega_{k} \end{array}$$

When SPA is applied in efficacy assessment, the results are subordinated to the principle of maximal connection degree, which means the maximal CD represents the CD of total indices. For a sample, assume d=max {a, b1, b2.* *.* *.* *b_k-2_, c} (k≥3). Total CD *φ*=a +b_1_i_1_+b_2_i_2_+* *. * *. * *.* *+b_k-2_i_k-2_+cj. Then through operation above, the efficacy grade should be d.

In this research, we set the SPA as a 5-element pattern; then, the general form of CD was *φ* =a+b_1_i_1_+b_2_i_2_+b_3_i_3_+cj, naming the 5 corresponding uncertain factors as unimportance (I), low importance (II), medium importance (III), high importance (IV), and higher importance (V). The certainty degree of the concept can be as connection degree; then SPA can be used to aggregate of quantitative with certain degree [15].

### 2.2. Cloud Model

Actually, in SPA method, each weight of indexes differs from each other in the efficacy assessment. The cloud model (CM) can embody uncertainly of the concept which is composed of cloud droplets that are random values and its degree can express the fuzziness; moreover, it could be more effective in achieving the efficacy assessment [15]. Cloud model was applied to analyze the weight of index used in the SPA. Conduct an efficacy assessment of diabetic ulcers by using CM-SPA.

Assume the given universe of discourse named U, and x represents any of elements ∈ U. If x fulfill the concept C, which is the correspondence between U and C(x) ∈[0,1], ‘x' seems to be a stochastic event with stable trends; therefore, distribution of x in U is a cloud, and each x is called a cloud drop.

(4) μ:U→0,1∀x∈Ux→μx

Concept C= (Ex, En, He) is clearly defined to characterize the uncertainty in CM. Among these concepts, expected value, entropy, and hyper entropy are described as Ex, En, and He, respectively. Ex denotes the most representative of this qualitative statement in the number of domain space which reflects the position of the center of gravity. En reveals the relevance of ambiguity and randomness. He is an uncertainty measure, the entropy of entropy, which reflects the small degree of certainty of cohesion of all points representing the linguistic value in the domain interrogation; that is, the degree of aggregation of cloud droplets. C= (Ex, En, He) is the qualitative concept in U which is the quantitative universe of discourse. Assume that a measurable value x (∀x ∈ U) is random for C and x fulfills x~N (Ex, En^′2^). In the same, En′ is also a stochastic instantiation and fulfills EN'~N (En, He^2^). Ex satisfies the following [8, 16].

(5) $$\begin{array}{c} \mu =e^{{\left(x-Ex\right)}^{2}/2{\left({En}^{\prime }\right)}^{2}} \end{array}$$

**Figure 1:**
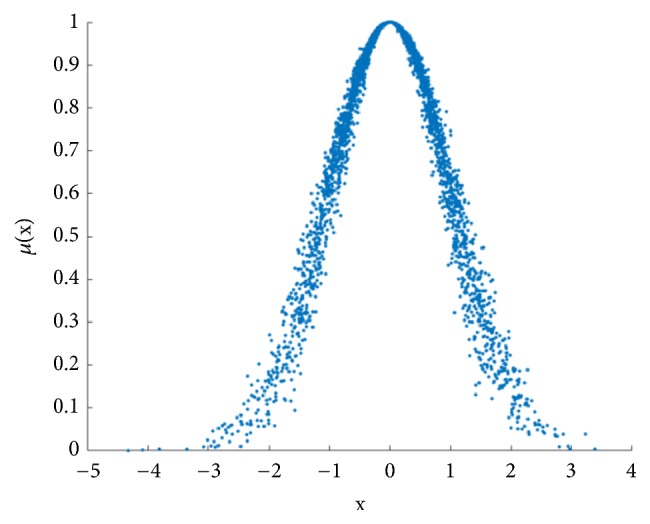
An example of normal cloud. Assume a normal cloud and let Ex, En, and He be 5, 1, and 0.1, respectively. Meanwhile, the amounts of cloud drops are set as 1000. Normal cloud images can be used as a reference standard for subsequent images.

Set Ex, En, and He as 5, 1, and 0.1, respectively, to assume the normal cloud which is operated by the forward cloud generator (Figure 1). Regarding the cloud drops that contribute to the qualitative concepts, they are mainly focused on [Ex-3En, Ex+3En]. Contribution of cloud drops outside [Ex-3En, Ex+3En] can be ignored. The definition is called the ‘3En rule' [16].

In order to represent the concept in CM, the confusion degree is introduced (see (6)). It needs to be pointed out that if the confusion value is greater than or equal to (but not less than) 1, then the cloud will be ‘fog', which means concepts need to be given again.

(6) $$\begin{array}{c} \text{confusion}\;\;degree=\frac{3He}{En} \end{array}$$

There is a multitude of influencing factors of diabetic ulcer. Experts on the different symptoms of the disease about the influence mechanism of the process of generation and development have not yet been fully revealed, and the problem of disease assessment has both fuzziness and randomness. By introducing the cloud theory to study the relationship between fuzziness and randomness, the problem of quantitative evaluation of diabetic ulcer can be well solved.

### 2.3. CM-SPA

In the article, CM was introduced into SPA to improve methods to calculate index weight, which is defined as the cloud weight. In traditional methods, input values of SPA are linguistic variables summed up from experts. However, how to reflect the uncertainty of linguistic variables is still an issue for the great influence of efficacy assessment results. A solution is cloud weight, which replaces the traditional weight for indices, and the cloud descriptors, which represent the efficacy assessment result. Detailed steps are shown below.

(1) Each index is evaluated by the linguistic variables. Evaluation criteria are shown in Table 1; then normalized results are shown below.

(7) $$\begin{array}{c} X_{kl}=\frac{x_{kl}}{\sum_{k=1}^{r}x_{kl}} \end{array}$$

In (7), x_kl_ represents the evaluations of index k made by expert 1 and r is amounts of indices, so that X_kl_ represents the normalized value of x_kl_.

(2) By obtaining the evaluations for index weight, the qualitative linguistic variables are transformed into quantitative values, expressed by the cloud descriptors Ex, En, and He (see (8)). Then, the cloud weight of index k, *ω*(C)k=(Ex_k_, En_k_, He_k_) is calculated below.

(8) Exk=1N∑l=1NXklEnk=π2×1N∑l=1NXkl−ExkHek=1N∑l=1NXkl−Exk2−Enk2

(3) As the cloud weight of the index was given, CCD, the total CD based on CM, can be calculated in the same way mentioned in SPA by (2) and (3) [17].

(9) ExsEx1⊕Ex2⊕…⊕Exk=Ex1+Ex2+…ExkEnsEn1⊕En2⊕…⊕Enk=En12+En22+…Enk2HesHe1⊕He2⊕…⊕Hek=He12+He22+…Hek2

(4) Final efficacy assessment results are shown in (9) which is made up of several normal clouds. In general, if the SPA model contains several elements, there will be the same number normal clouds shown in the final efficacy assessment results. The uncertain factors assessment result is confirmed according to the ‘3En rule' and the maximal connection degree principle as previously mentioned.

## 3. Case Study

In this study, the key factors of diabetic ulcer were taken as the background. According to previous clinical observation, we consider the impact of diabetes ulcer prognosis indicators are wound area, wound depth, exudates color, exudates volume, necrotic tissue area, new granulation and epithelial tissue color, new granulation and epithelial tissue area, wound skin temperature, wound skin color, and pain [18]. Afterwards, preclinical studies of three patients [18] which have been approved by the Ethics Committee of Yueyang Hospital of Integrated Traditional Chinese and Western Medicine Affiliated to Shanghai University of traditional Chinese medicine (protocol 2016061) conducted an importance assessment using CM-SPA. SPA was set as a 5-element model; moreover, general type of CD was *φ* = a+b_1_i_1_+b_2_i_2_+b_3_i_3_+cj; then 5 corresponding uncertain factors were named as unimportance (I), low importance (II), medium importance (III), high importance (IV), and higher importance (V).

10 experts were invited to assess their importance based on Table 1, and their introductions were displayed in Table 2. Ex, En, and He were computed by (7) through (8). The judgment results and cloud weight of each uncertain factors are displayed in Tables 3 and 4. Based on the SPA definition that is mentioned previously, the evaluations for each importance grade were confirmed by Table 5 according to severity score for diabetic foot ulcers [19] and Table 6 is based on the patients' symptoms after the treatment with traditional Chinese medicine. The results are shown in Table 7.

## 4. Results

**Figure 2:**
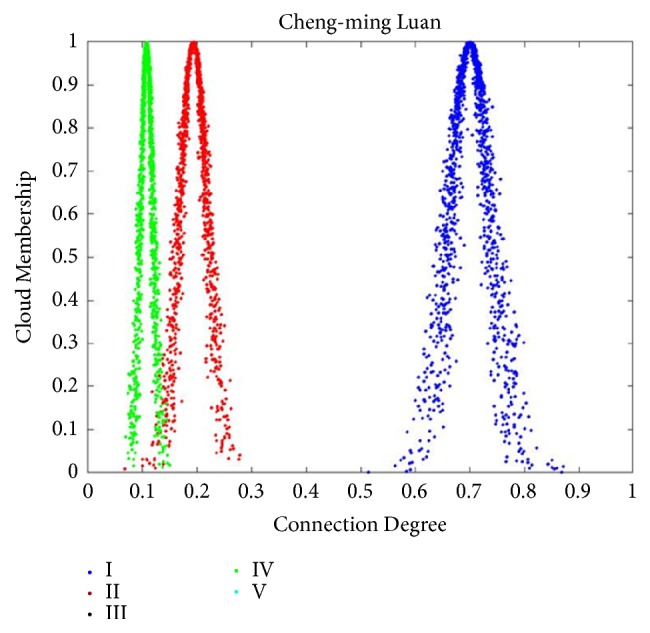
Normal clouds of CCD for Cheng-ming Luan. Suppose the number of cloud droplets is 1000; the normal cloud corresponding to each evaluation level is generated using the results of CCD operations and algorithms written on the Matlab platform, and the cloud droplets corresponding to each level are shown in the legend. According to the maximum connectivity principle and ‘3En rule', the grade of Cheng-ming Luan completely belonged to grade I and the efficacy grade of Cheng-ming Luan was very high efficacy.

**Figure 3:**
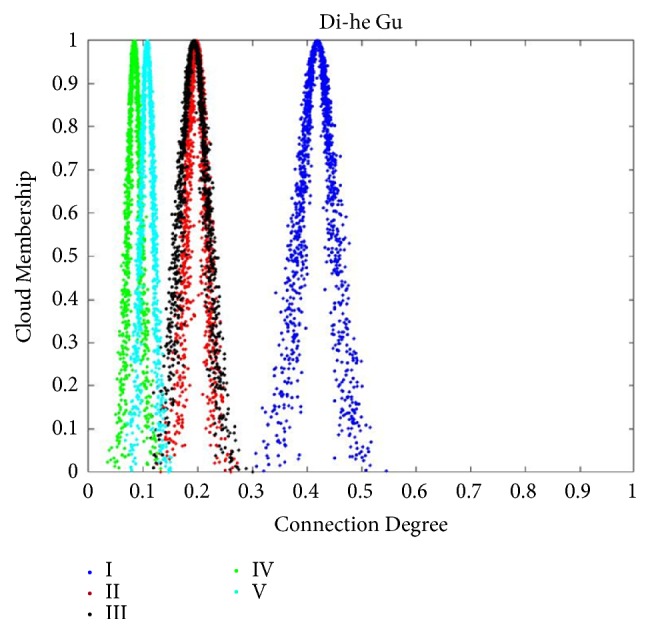
Normal clouds of CCD for Di-he Gu. Suppose the number of cloud droplets is 1000; the normal cloud corresponding to each evaluation level is generated using the results of CCD operations and algorithms written on the Matlab platform, and the cloud droplets corresponding to each level are shown in the legend. According to the maximum connectivity principle and ‘3En rule', the grade of Di-he Gu completely belonged to grade I and the efficacy grade of Cheng-ming Luan was very high efficacy.

**Figure 4:**
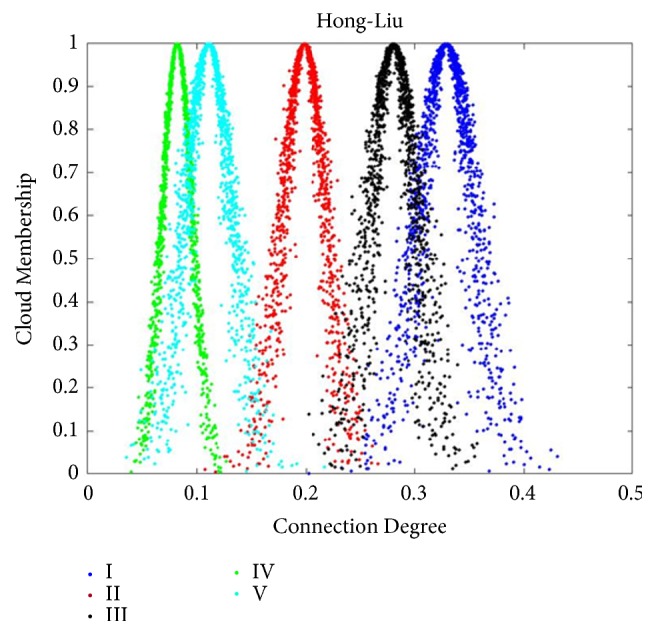
Normal clouds of CCD for Hong-Liu. Suppose the number of cloud droplets is 1000; the normal cloud corresponding to each evaluation level is generated using the results of CCD operations and algorithms written on the Matlab platform, and the cloud droplets corresponding to each level are shown in the legend. According to the maximum connectivity principle and ‘3En rule', the grade of Hong-Liu was between grade I and grade III and closer to grade I. Hence, the efficacy grade of Hong-Liu was between very high efficacy and middle efficacy and closer to very high efficacy.

After obtaining the evaluations of each importance grade of the three patients, the CCD was computed by using (9), and the calculation results were shown in Table 8. Results of importance assessment analyzed by CM-SPA as shown in Figures 2, 3, and 4. In consideration of constant values of indices weights, AHP is most appropriate which is extensively used in the determination of SPA index weights. In order to validate the comparability of result, the same data was employed by AHP based SPA. The following is a brief description of the determination of indices weights obtained by AHP.

(1) Firstly, efficacy assessment for diabetic ulcers was treated as the overall goal of AHP. Then, the two basic factors for evaluating the efficacy of diabetic ulcer patients, the degree of wound healing and hypertrophic scar, were taken as the intermediate factors of analytic hierarchy process (AHP). Therefore, these ten evaluation indexes were taken as the evaluation criteria of analytic hierarchy process (AHP).

(2) Pairwise comparison matrix was used to judge both importance of criteria to middle factors and middle factors to the overall goal [16]. The matrix reflected the pairwise comparison and was compared on a scale of 1/9-9. The judgment matrix of wound healing degree and hypertrophic scar to the overall target was expressed as matrix M1 (see (10)), and the judgment matrix of wound healing degree and 10 evaluation indexes of hypertrophic scar were expressed by matrices M2 and M3 (see (11)-(12)), respectively. P1 and p2 indicated the degree of wound healing and hypertrophic scar, respectively, and *α* denoted the pairwise comparison result. The pairwise comparison results in the matrix summarized from wound-based severity score for diabetic foot ulcers [19].

(10) M1αp1p1αp1p2αp2p1αp2p2=13131

(11) M2αk1k1αk1k2αk1k3αk1k4αk1k5αk1k6αk1k7αk1k8αk1k9αk1k10αk2k1αk2k2αk2k3αk2k4αk2k5αk2k6αk2k7αk2k8αk2k9αk2k10αk3k1αk3k2αk3k3αk3k4αk3k5αk3k6αk3k7αk3k8αk3k9αk3k10αk4k1αk4k2αk4k3αk4k4αk4k5αk4k6αk4k7αk4k8αk4k9αk4k10αk5k1αk5k2αk5k3αk5k4αk5k5αk5k6αk5k7αk5k8αk5k9αk5k10αk6k1αk6k2αk6k3αk6k4αk6k5αk6k6αk6k7αk6k8αk6k9αk6k10αk7k1αk7k2αk7k3αk7k4αk7k5αk7k6αk7k7αk7k8αk7k9αk7k10αk8k1αk8k2αk8k3αk8k4αk8k5αk8k6αk8k7αk8k8αk8k9αk8k10αk9k1αk9k2αk9k3αk9k4αk9k5αk9k6αk9k7αk9k8αk9k9αk9k10αk10k1αk10k2αk10k3αk10k4αk10k5αk10k6αk10k7αk10k8αk10k9αk10k10=113133113553135533577113133113551315131113131331315131113131331131331135511313311355131513111313153151715131315151511151715131315151311

(12) M3αk1k1αk1k2αk1k3αk1k4αk1k5αk1k6αk1k7αk1k8αk1k9αk1k10αk2k1αk2k2αk2k3αk2k4αk2k5αk2k6αk2k7αk2k8αk2k9αk2k10αk3k1αk3k2αk3k3αk3k4αk3k5αk3k6αk3k7αk3k8αk3k9αk3k10αk4k1αk4k2αk4k3αk4k4αk4k5αk4k6αk4k7αk4k8αk4k9αk4k10αk5k1αk5k2αk5k3αk5k4αk5k5αk5k6αk5k7αk5k8αk5k9αk5k10αk6k1αk6k2αk6k3αk6k4αk6k5αk6k6αk6k7αk6k8αk6k9αk6k10αk7k1αk7k2αk7k3αk7k4αk7k5αk7k6αk7k7αk7k8αk7k9αk7k10αk8k1αk8k2αk8k3αk8k4αk8k5αk8k6αk8k7αk8k8αk8k9αk8k10αk9k1αk9k2αk9k3αk9k4αk9k5αk9k6αk9k7αk9k8αk9k9αk9k10αk10k1αk10k2αk10k3αk10k4αk10k5αk10k6αk10k7αk10k8αk10k9αk10k10=1311313113113131311317171131151131115151533713153773371311337911151511111133113131113191111517131311713551717197113113131911111

(3) Finally, indices weights were computed based on the above matrices while calculation results are shown in Table 9.

After obtaining the AHP based indices weights, CDs were calculated with (3); see Tables 7 and 9. Final results of CD were shown in Table 10. According to the maximum connectivity principle and ‘3En rule', as mentioned earlier, in Figure 2, the CDs of grades I, II, and IV were mainly focused on [0.5873, 0.8124], [0.1156,0.2700], and [0.0723,0.1423], respectively. There was no intersection between grade I and the other grades. In other words, the grade of Cheng-ming Luan completely belonged to grade I and the efficacy grade of Cheng-ming Luan was very high efficacy. In Figure 3, the CDs of grades I, II, III, IV, and V were mainly focused on [0.3267, 0.5107], [0.1437,0.2514], [0.1156,0.2700], [0.0475,0.1198], and [0.0723,0.1423], respectively. There was no intersection between grade I and the other grades. In other words, the grade of Di-he Gu completely belonged to grade I and the efficacy grade of Di-he Gu was very high efficacy. In Figure 4, the CDs of grades I, II, III, IV, and V were mainly focused on [0.2510,0.4063], [0.1380,0.2589], [0.2152,0.3449], [0.0405,0.1236], and [0.0456,0.1758], respectively. It can be concluded that the intersection scope of grade I and grade III was the maximum; thus, the relevance of grade I to grade III was closer than for other grades. Then, it was summarized that the grade of Hong-Liu was between grade I and grade III and closer to grade I. That is to say, the efficacy grade of Hong-Liu was between very high efficacy and middle efficacy and closer to very high efficacy.

In Table 10, similar results were obtained by AHP that after the treatment of traditional Chinese medicine; the curative effect ratings of the three people all belonged to grade I according to the maximum connectivity principle that also confirms the scientific nature of SPA-CM to a certain extent.

## 5. Discussion

Diabetic ulcer is a system of uncertainty such as wound area, wound depth, and exudate color [18]. At present, there are many methods that can be used to evaluate the curative effect of diabetic ulcer, such as risk index method, analytic hierarchy process (AHP), gray relational analysis, and bow tie model [16, 20]. These evaluation methods have their own advantages and disadvantages; disadvantages are mainly for the subjectivity of weight assignment, ignoring the uncertainty of the evaluation process.

Based on the above research status, we intend to solve the subjective problem of weight assignment of uncertain factors in the evaluation system and combined with the mathematical method to study uncertain factors to carry out comprehensive evaluation of diabetic ulcer patients. At present, SPA has been used in a host of fields of efficacy assessment. For instance, SPA based layer of protection analysis has been applied in quantitative risk assessment [21]. Besides, SPA has been used to evaluate the emergency response capacity of large airports, making sure the factors and mechanism of airport vulnerability and emergency response capacity on airport flexibility, which has improved the resilience of airports [22, 23].

In this paper, an evaluation method combining cloud model (CM) with set pair analysis (SPA) to deal with uncertain factors is adopted. This method uses the fuzziness and randomness of cloud model to determine cloud weight and reduces the subjectivity of assignment. On this basis, combined with set pair analysis, the comprehensive evaluation of diabetic ulcer patients was carried out, and the comprehensive evaluation set pair cloud model was obtained, so as to determine the curative effect rating of the evaluated object and to show it intuitively in the form of cloud droplets.

## 6. Conclusions

(1) In the paper, cloud model (CM) was used to improve set pair analysis (SPA). The cloud weight should be used in the analysis of SPA. Different from the traditional index weights of SPA as constant values, cloud weights were defined as the cloud descriptors which can reflect the fuzziness and randomness of experts' judgments. Thus the subjectivity of index weight determination can be avoided by using CM-SPA.

(2) The obtained assessment results by AHP based SPA were not reasonable and precise enough due to the influences of other parameters of the CD were neglected. For example, in regard to the assessment result of Hong-Liu, though assessment values of grade I (0.3667) and grade III (0.3614) were almost equal, the assessment result can only confirmed to be grade I due to the maximal connection degree principle while CM-SPA came to the conclusion between grade I and grade III and closer to grade I. In addition, with the exception of the maximal parameter of the CD, a slice of other parameters were not considered in confirming assessment results even though values of them were not ‘0'. In contrast to CM-SPA, efficacy evaluation results of the classical method AHP can only be reflected by the obviously different efficacy level. For the CM-SPA, slight variation of efficacy treatments will be caught for its smaller precision, and the evaluation results will be more accurate and realistic.

(3) For the CM-SPA, further and complete assessments should be made based on the randomness and fuzziness of CM; the assessment results will be more precise and reasonable.

## Figures and Tables

**Table 1:** Judgment criterion of experts for index weight. The table shows the expert judgment criteria of index weight. The important level is divided into five levels from very unimportant to very important, and the corresponding weight number is gradually increased from 0 to 10. Experts rate the importance of different symptoms.

Linguistic Variables Level	Value Range
Very important	(8, 10]
Important	(6, 8]
Middle important	(4, 6]
Unimportant	(2, 4]
Very unimportant	(0, 2]

**Table 2:** Introduction of the experts. The table provides an introduction to the expert, consisting of 2 students, 3 doctors-in-charge, 4 associate professors, and 3 professors, with work experience ranging from 2 to 40 years.

	Professional position	Education background	Experience(years)
Expert1	Student	Bachelor	2

Expert2	Student	Master	4

Expert3	Doctor-in-charge	Master	10

Expert4	Doctor-in-charge	Master	10

Expert5	Doctor-in-charge	PhD	15

Expert6	Associate Professor	Master	22

Expert7	Associate Professor	PhD	25

Expert8	Professor	PhD	29

Expert9	Professor	PhD	35

Expert10	Professor	PhD	40

**Table 3:** Results of experts' judgments regarding the importance of assessment indices. The corresponding meanings of the K value are expressed as follows. K1: wound area; K2: wound depth; K3: exudates color; K4: exudates volume; K5: necrotic tissue area; K6: new granulation and epithelial tissue color; K7: new granulation and epithelial tissue area; K8: wound skin temperature; K9: wound skin color; K10: pain.

	K1	K2	K3	K4	K5	K6	K7	K8	K9	K10
Expert1	6	10	10	8	8	9	8	7	8	5
Expert2	10	9	8	6	8	9	9	8	7	5
Expert3	7	10	6	5	8	9	8	5	5	5
Expert4	9	8	9	6	7	9	9	7	5	6
Expert5	10	10	8	8	9	8	7	7	7	6
Expert6	8	8	5	5	5	5	5	4	4	5
Expert7	6	7	6	6	5	7	8	8	7	7
Expert8	7	9	8	7	7	7	8	6	6	7
Expert9	10	7	9	9	7	9	9	9	7	7
Expert10	7	9	8	5	8	7	7	7	5	6

**Table 4:** Cloud weight of each index. Ex, En and He denote the expected value, entropy, and hyper entropy, respectively. Ex is the expected value of the cloud drop that can represent the qualitative concept. En reflects the dispersion degree of cloud drops, which also determines the certainty of cloud drops. He is the entropy of En. It reveals the uncertainty measurement of En that is used to settle confusion degree (see (6)). For the commonsense concept, He is smaller when the acceptance degree is higher. In the same way, He will be bigger if the concept cannot reach an agreement. After the test of (6), the distribution of Ex, En, and He in Table 4 is in accordance with the shape of cloud.

Index	Ex	En	He
Wound area(K1: cm^2^)	0.11073	0.0217	0.00486
Wound depth(K2: cm)	0.12116	0.01886	0.00515
Exudates color(K3)	0.10537	0.01384	0.00412
Exudates volume(K4: layers of gauze wetted)	0.0891	0.01365	0.00451
Necrotic tissue area (K5: %)	0.09907	0.01335	0.00348
New granulation & epithelial tissue color(K6)	0.10844	0.01164	0.00255
New granulation & epithelial tissue area (K7:%)	0.10733	0.01166	0.00280
Wound skin temperature(K8)	0.09309	0.01464	0.00168
Wound skin color(K9)	0.08363	0.01205	0.00308
Pain(K10:VAS)	0.08207	0.01385	0.00154

**Table 5:** Classification of grade according to severity score for diabetic foot ulcers.

Index	Grade				
	I	II	III	IV	V
Wound area(K1: cm^2^)	0	1-4	4-9	9-16	>16

Wound depth(K2: cm)	0-1	1-2	2-3	3-4	>4

Exudates color(K3)	Transparent	Red	Yellow	Green	Black

Exudates volume(K4: layers of gauze wetted)	0-4	5-8	9-12	13-16	>16

Necrotic tissue area (K5: %)	0-20	21-40	41-60	61-80	81-100

New granulation & epithelial tissue color(K6)	Bright red	Red	Light red	Pink	Pale

New granulation & epithelial tissue area (K7:%)	81-100	61-80	41-60	21-40	0-20

Wound skin temperature(K8)	Normal	Slightly hot	Hot	Pretty hot	Scorching hot

Wound skin color(K9)	Normal	Reddish	Red	Bright red	Dark red

Pain(K10:VAS)	0-2	3-4	5-6	7-8	9-10

**Table 6:** Index data of three diabetic ulcer patients from preclinical studies after TCM treatment.

Index	Cheng-ming Luan	Di-he Gu	Hong-Liu
Wound area(K1: cm^2^)	1	6	35

Wound depth(K2: cm)	0.5	0.3	0.4

Exudates color(K3)	Transparent	Transparent	Red

Exudates volume(K4: layers of gauze wetted)	3	7	11

Necrotic tissue area (K5: %)	0	0	10

New granulation & epithelial tissue color(K6)	Bright red	Red	Bright red

New granulation & epithelial tissue area (K7:%)	24	0	42

Wound skin temperature(K8)	Normal	Normal	Slightly hot

Wound skin color(K9)	Normal	Bright red	Red

Pain(K10:VAS)	3	5	7

**Table 7:** Evaluations for each important grade by combining the data in Tables 5 and 6.

Cheng-ming Luan						Di-he Gu					Hong-Liu				
	I	II	III	IV	V	I	II	III	IV	V	I	II	III	IV	V
K1	0	1	0	0	0	0	0	1	0	0	0	0	0	0	1

K2	1	0	0	0	0	1	0	0	0	0	1	0	0	0	0

K3	1	0	0	0	0	1	0	0	0	0	0	1	0	0	0

K4	1	0	0	0	0	0	1	0	0	0	0	0	1	0	0

K5	1	0	0	0	0	1	0	0	0	0	1	0	0	0	0

K6	1	0	0	0	0	0	1	0	0	0	1	0	0	0	0

K7	0	0	0	1	0	0	0	0	0	1	0	0	1	0	0

K8	1	0	0	0	0	1	0	0	0	0	0	1	0	0	0

K9	1	0	0	0	0	0	0	0	1	0	0	0	1	0	0

K10	0	1	0	0	0	0	0	1	0	0	0	0	0	1	0

**Table 8:** Calculation results of CCD. Ex, En, and He denote the expected value, entropy, and hyper entropy, respectively. Ex is the expected value of the cloud drop that can represent the qualitative concept. En reflects the dispersion degree of cloud drops, which also determines the certainty of cloud drops. He is the entropy of En. It reveals the uncertainty measurement of En that is used to settle confusion degree (see (6)). For the commonsense concept, He is smaller when the acceptance degree is higher. In the same way, He will be bigger if the concept cannot reach an agreement. After the test of (6), the distribution of Ex, En, and He in Table 8 is in accordance with the shape of cloud.

	Cheng ming Luan			Di-he Gu			Hong-Liu		
	Ex	En	He	Ex	En	He	Ex	En	He
I	0.6999	0.0375	0.0097	0.4187	0.0307	0.0076	0.3287	0.0258	0.0067
II	0.1928	0.0257	0.0051	0.1975	0.0179	0.0052	0.1985	0.0201	0.0045
III	0	0	0	0.1928	0.0257	0.0051	0.2801	0.0216	0.0061
IV	0.1073	0.0117	0.0028	0.0836	0.012	0.0031	0.0821	0.0138	0.0015
V	0	0	0	0.1073	0.0117	0.0028	0.1107	0.0217	0.0049

**Table 9:** Indices weights obtained by AHP. The table is the weight of ten indexes calculated according to the matrix, in which the judgment matrix of the criterion layer to the target layer and the judgment matrix of the scheme layer to the criterion layer satisfy the consistency.

	K1	K2	K3	K4	K5	K6	K7	K8	K9	K10
weight	0.0658	0.0916	0.1163	0.1706	0.1996	0.0755	0.0716	0.0456	0.1191	0.0443

**Table 10:** Calculation results of CD. The table is the decision set calculated based on (3) and Tables 7 and 9. The curative effect ratings of Cheng-ming Luan, Di-he Gu and Hong-Liu all belonged to grade I according to the maximum connectivity principle.

	Cheng-ming Luan	Di-he Gu	Hong-Liu
I	0.8183	0.4531	0.3667

II	0.1101	0.2461	0.1619

III	0.0000	0.1101	0.3614

IV	0.0716	0.1191	0.0443

V	0.0000	0.0716	0.0658

## Data Availability

All of the data used to support the findings of this study are available from the corresponding author upon request.

## Supplementary Materials

Supplementary Materials We used Metlab software to write code to calculate the Ex, En, and He of SPA-CM by applying (8) and (9) and the weight of AHP's ten metrics. The supplementary material is the calculation code of Metlab.
