# Supplementary material for: Application of a Cloud Model-Set Pair Analysis in Efficacy Assessment for Diabetic Ulcers
Source: Evid Based Complement Alternat Med. 2019 Jun 18;2019:8450397. doi: 10.1155/2019/8450397 (PMC6604411; doi:10.1155/2019/8450397)
Supplement: Supplementary Materials — We used Metlab software to write code to calculate the Ex, En, and He of SPA-CM by applying (8) and (9) and the weight of AHP's ten metrics. The supplementary material is the calculation code of Metlab. [file 8450397.f1.docx]

**Calculation process raw data**

**1、We uesd metlab software to write code to calculate the Ex,En,He of SPA-CM by applying Eq8 and Eq9, the specific content is shown as follows**

**1. 1 The code used to calculate the Ex,En,He in table4:**

D=xlsread(path1); % matrix D is the result table of experts' judgment on the importance of evaluation indexes.

sum=zeros(size(D,1),1);

E=zeros(size(D,1),size(D,2)); % matrix E is a 0 matrix of the same class as matrix D

for i=1:size(D,1)

for j=1:size(D,2)

sum(i,1)=sum(i,1)+D(i,j);

end

for j=1:size(D,2)

E(i,j)=D(i,j)/sum(i,1); % operation standardization result

end

end

function [x, y, Ex, En, He] = cloud_T(E(:,ii),N) % The size of ii is the same as the number of columns of matrix D

Ex = mean(E(:,ii)); % calculate the mean value of standardized results, that is, the expected value Ex

En = mean(abs(E(:,ii) - Ex)).*sqrt(pi./2); % calculating the value of En by applying Eq 8

He = sqrt(abs(var(E(:,ii),0) - En.^2)); % calculating the value of He by applying Eq 8

**1.2 The code used to calculate the Ex,En,He in table8:**

A=xlsread(path4,'sheet1'); % Matrix A is the content of evaluations for each important grade in Table7

R=xlsread(path2); % Matrix R is the content of cloud weight of each index in Table4

result2=zeros(5,3); % Defining an 0 Matrix of 5*3

for j = 1: 5

Ex=0;

En2=0;

He2=0; % Initial value definition for Ex、En2、He2

for i=1:size(R)

if A(i,j)==1

Ex=Ex+R(i,1); % Calculation of the value of Exs in Eq 9 in combination with Table7

En2=En2+(R(i,2))^2; % Calculation of the sum of squares of En in Eq 9 combined with Table7

He2=He2+(R(i,3))^2; % Calculation of the sum of squares of He in Eq 9 combined with Table7

end

end

En = sqrt(En2); % Open the root number to En2 and get the value of En

He = sqrt(He2); % He value is obtained by open the root number to He2, and finally the operation result of the CCD.

End

**2、 Code used to calculate the weight of AHP's ten metrics**

Aa=[1,1/3;3,1]; % Judgment Matrix M1 of input Criterion layer to Target layer

B1=xlsread(path5);

B2=xlsread(path6); % Call the judgment matrix M2, M3 of the entered scheme layer to the criterion layer

BS = [B1,B2]; % Combine M2 and M3 into a matrix

m = length(B1);

n = length(Aa); % Gets the number of rows of three matrices

[Wa,LA] = eig (Aa); % Calculation of Eigenvectors WA and Eigenroots LA of M1

WA=Wa(:,1)/sum(Wa(:,1)); % Eigenvector normalization

for k = 1:n % Calculation of Eigenvectors WK and Eigenroot LK of M2 and M3

[WB,LK] = eig( BS(1:10,(k-1)*m+1:(k-1)*m+10) );

WK(:,k)= WB(:,1)/sum(WB(:,1));

end

E = WK * WA;

disp(' Scheme layer combination weight vector ');

disp(E); % Output scheme layer combination weight vector

Code for calculating CD results

A=xlsread(path4,'sheet1'); %Matrix A is the first patient's ' Evaluations for each important grade'

result3=zeros(5,3); % Define a 0 matrix of 5*3

for j = 1: 5

single=E.*A(:,j); % Multiplies the values in the scheme-level combination weight vector by each column of the table7 in turn

result3(j,1)=sum(single,1); % The single product is added to obtain an operation result of CD.

end

B=xlsread(path4,'sheet2'); %Matrix B is second patient's ' Evaluations for each important grade'

grade for j = 1: 5

single=E.*B(:,j);

result3(j,2)=sum(single,1);

end

C=xlsread(path4,'sheet3'); %Matrix C is third patient's ' Evaluations for each important grade'

grade for j = 1: 5

single=E.*C(:,j);

result3(j,3)=sum(single,1);

end

xlswrite(path,E,'Sheet1') % Save calculation results

xlswrite(path,result3,'Sheet2')
